# Viper and Cobra Venom Neutralization by Alginate Coated Multicomponent Polyvalent Antivenom Administered by the Oral Route

**DOI:** 10.1371/journal.pntd.0003039

**Published:** 2014-08-07

**Authors:** Sourav Bhattacharya, Mousumi Chakraborty, Piyasi Mukhopadhyay, P. P. Kundu, Roshnara Mishra

**Affiliations:** 1 Department of Physiology, University of Calcutta, Kolkata, India; 2 Centre for Research in Nanoscience and Nanotechnology, University of Calcutta, Kolkata, India; 3 Department of Polymer Science and Technology, University of Calcutta, Kolkata, India; University of Newcastle, Australia

## Abstract

**Background:**

Snake bite causes greater mortality than most of the other neglected tropical diseases. Snake antivenom, although effective in minimizing mortality in developed countries, is not equally so in developing countries due to its poor availability in remote snake infested areas as, and when, required. An alternative approach in this direction could be taken by making orally deliverable polyvalent antivenom formulation, preferably under a globally integrated strategy, for using it as a first aid during transit time from remote trauma sites to hospitals.

**Methodology/Principal Findings:**

To address this problem, multiple components of polyvalent antivenom were entrapped in alginate. Structural analysis, scanning electron microscopy, entrapment efficiency, loading capacity, swelling study, *in vitro* pH sensitive release, acid digestion, mucoadhesive property and venom neutralization were studied in *in vitro* and *in vivo* models. Results showed that alginate retained its mucoadhesive, acid protective and pH sensitive swelling property after entrapping antivenom. After pH dependent release from alginate beads, antivenom (ASVS) significantly neutralized phospholipaseA_2_ activity, hemolysis, lactate dehydrogenase activity and lethality of venom. In *ex vivo* mice intestinal preparation, ASVS was absorbed significantly through the intestine and it inhibited venom lethality which indicated that all the components of antivenom required for neutralization of venom lethality were retained despite absorption across the intestinal layer. Results from *in vivo* studies indicated that orally delivered ASVS can significantly neutralize venom effects, depicted by protection against lethality, decreased hemotoxicity and renal toxicity caused by russell viper venom.

**Conclusions/Significance:**

Alginate was effective in entrapping all the structural components of ASVS, which on release and intestinal absorption effectively reconstituted the function of antivenom in neutralizing viper and cobra venom. Further research in this direction can strategize to counter such dilemma in snake bite management by promoting control release and oral antivenom rendered as a first aid.

## Author Summary

Antivenom, the only effective therapy against snake bite in practice, is successful in controlling mortality in developed countries, but not in developing countries. Unavailability of antivenom at the proper time and place of snake bite in developing countries is a major factor in this account, which results not only from production deficit but also from dependence on hospitals located too faraway for intravenous administration. It lengthens the period between bite and treatment, and thereby worsens the outcome. To make antivenom available immediately after bite, we need to develop an oral formulation which, by its property of controlled release, can supply antivenom as first aid until further hospitalization. In this work, multiple components of antivenom were entrapped in alginate, an economic, biodegradable polymer, which retained the functional property of the antivenom even after intestinal absorption and showed *in vivo* and *in vitro* venom neutralization effects. This study promises the development of an effective first aid against snake envenomation, thereby increasing chances of survival of the victim.

## Introduction

The World Health Organization (WHO) [1] has enlisted snake bite as one of the neglected tropical diseases. About 5.5 million snake bites resulting in about 40 thousand amputations and 20 to 125 thousand deaths have greater mortality than that from other neglected tropical diseases viz. dengue, hemorrhagic fever, cholera, leishmaniasis, schistosomiasis, Japanese encephalitis, and Chagas' disease [2]. In India the magnitude of mortality is grave, at about 0.47% of total deaths [3]. Although antisnake venom serum (ASVS) is effective in keeping the mortality low in developed countries, in developing countries the same solution is rendered ineffective by several factors typical to neglected tropical diseases. Brown [4] has encountered lack of effective, safe and affordable therapy in developing countries while, Warrel [5], suggested improving production and clinical use of antivenom. Critical analysis of high mortality from snake bite not only indicates shortcomings of ASVS alone, but also insufficiency of infrastructure in snake infested developing countries. Prognosis depends on early ASVS administration which needs hospitalization for intravenous delivery and for treating hypersensitive reaction from ASVS. Transit time to hospital thus is an important determinant factor in outcome as bites mostly occur in remote places. In most of the developing countries remoteness, cost and heat-instability of ASVS are major contributing factors of the inaccessibility of ASVS [6]. Remoteness increases the cost further than the production cost by adding to the cost of distribution, storage, administration and of providing infrastructure for reaching remote areas. So, making ASVS effectively available is a critical factor which requires globally integrated knowledge based strategy [7]. An approach to address the problem of remoteness suggested use of Geographical Information Systems for cost effective utilization of ASVS [8]. In this work we have elaborated another approach to develop readily available and orally deliverable polyvalent ASVS formulation for use it as first aid by local health practitioners during transit to hospital. This approach can change the prognosis of snake bite by preventing the irreversible damage from venom resulting during transit time. Oral and controlled ASVS delivery as first aid prior to hospitalization can change the prognosis by multiple factors – 1. Use of easy-to-administer ASVS as first aid, 2. Less irreversible damage from venom during transit time 3. Less reliance on faith healers if treatment could be started immediately, 4. Less chance of adverse effect during transit due to controlled release properties of oral formulation. These factors together can make local and timely availability of ASVS feasible. Moreover, as a part of a global strategy, it can help in designing a global oral formulation for first aid.

Many approaches were taken for delivering protein drugs through oral route like the use of polymers [9]–[11], liposome based drug delivery [12], [13]or by using nanotechnology [14]–[16]. Present study was aim to encapsulate a drug which unlike insulin, BSA, or immunoglobulin, is a combination of multiple heterogenous proteins of different molecular weight and isoelectric pHs. Drug delivery research has not yet dealt with such problems where the components are not only multiple but also not studied individually. Therefore, we took alginate a biocompatible, biodegradable, non toxic polymer, known to encapsulate a wide variety of molecules [17], for bead preparation and studied, whether upon entrapment, the beads retain the advantages of alginate while the antivenom, retains its structural components which, on pH dependent release, preserve the capacity to neutralize venom activity.

## Methods

### Ethics statement

All animal experiments were approved by the Animal Ethics Committee of the University of Calcutta and were in accordance with the guidelines of the Committee for the Purpose of Control and Supervision of Experiments on Animal (CPCSEA), Government of India (IAEC Ref no:820/04/ac/CPCSEA.2010).

### Animals

Wistar strain male albino rats of about 9–12 weeks old (240±20 g) were used in the experiment. Animals were collected from Chakraborty and company, Calcutta, and housed under controlled environment (RT: 22±2°C, relative humidity: 60±5%, 12 h day/night cycle) with balanced diet and water *ad libitum*. All animal experiments were approved by the animal ethical committee, Department of Physiology, Calcutta University and were in accordance with the guideline of the committee for the purpose of control and supervision of experiments on animal (CPCSEA Ref no: 820/04/ac/CPCSEA.2010), Government of India.

### Collection and preparation of venom

Indian spectacle cobra (*Naja naja*) venom and Russell's viper (*Daboia russelii*) venom was gifted by Dr. Debanik Mukherjee, Field Biologist (Herpetology), Centre for Environmental Management of Degraded Ecosystems (CEMDE), University of Delhi, India. Venoms were lyophilized and stored at 4°C in amber colored bottle until further use. For the experiments, relevant venoms were weighed, dissolved in 0.9% saline and used at appropriate dilutions.

### Characterization of alginate ASVS beads

#### Preparation of alginate entrapped ASVS beads

**Figure 1:**
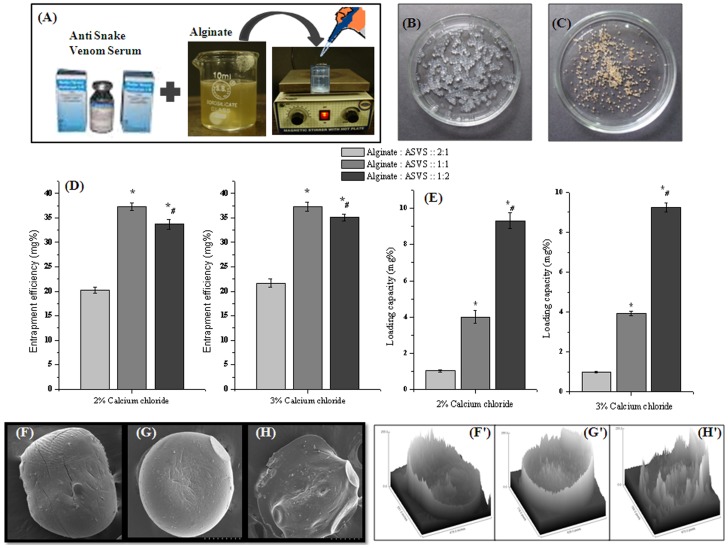
Preparation and characterization of alginate-ASVS beads. (A) Schematic diagram of the preparation of alginate entrapped ASVS beads; (B) Wet Algenate-ASVS beads; (C) Dry Algenate-ASVS beads. (D) Entrapment efficiency of alginate ASVS beads at different concentration ratio of alginate: ASVS:: 2∶1; alginate: ASVS :: 1∶1; alginate: ASVS :: 1∶2 in 2% and 3% calcium chloride solution. (E) Loading capacity of alginate entrapped ASVS beads at different concentration ratio of alginate: ASVS :: 2∶1; alginate: ASVS ::1∶1; alginate: ASVS ::1∶2 in 2% and 3% calcium chloride solution. Result showed as mean ± SEM, n = 6. *# *p*<0.05 was considered significant (*alginate: ASVS :: 2∶1 vs alginate: ASVS :: 1∶1; and alginate: ASVS :: 1∶2; # alginate: ASVS :: 1∶1 vs alginate: ASVS :: 1∶2). (F) SEM image of alginate: ASVS :: 2∶1 bead; (F′) Surface morphology analysis from SEM images of alginate: ASVS :: 2∶1 bead using image J software; (G) SEM image of alginate: ASVS :: 1∶1 bead; (G′) Surface morphology analysis from SEM images of alginate: ASVS :: 1∶1 bead using image J software; (H) SEM image of alginate: ASVS :: 1∶2 bead; (H′) Surface morphology analysis from SEM images of alginate: ASVS :: 1∶2 bead using image J software.

Aqueous solution of ASVS (VINS Bioproduct Ltd, India) was prepared and protein concentration of the solution was determined by Lowry et al [18] which is based on reaction of peptide bond with copper ion combined with aromatic amino acid residue oxidation. ASVS solution (250 µg protein/ml) was then mixed with 2% sodium alginate solution (w/v) in different ratio (alginate: ASVS:: 2∶1; alginate: ASVS:: 1∶1; and alginate: ASVS:: 1∶2) (v/v) on magnetic rotor. ASVS mixed alginate was then added drop wise to 2% and 3% calcium chloride solution for cross linking in rotating condition and kept for 15 minutes. After 15 minutes beads were washed thoroughly with distilled water and dried at room temperature (Fig. 1A, 1B, 1C).

#### Entrapment efficiency and loading capacity

After preparation of alginate entrapped ASVS beads remaining calcium chloride solution was recovered and concentration of free ASVS was determined as protein concentration by Lowry et al. 1951. Entrapment efficiency and loading capacity [19] was determined using the following formulae:
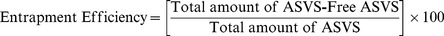


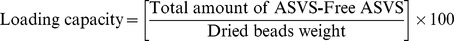



#### Structural analysis study using SEM

The morphological characteristics of alginate and alginate entrapped ASVS beads were examined by scanning electron microscopy (JSM-5900LV, JEOL, Japan). Beads were sputtered with gold and maintained at room temperature for complete dryness before the observation. Images were analyzed by Image J software to determine the surface characteristics.

#### Swelling study

Swelling study was done using 10 mg of alginate entrapped ASVS beads in 10 ml of HCl- KCl buffer (pH-1.2), Phosphate buffer saline (PBS) at pH- 6.8, 7.0 and 7.4 at 37°C simulating pH condition of stomach and intestine. All the solutions were then mixed at 100 rpm for 10 minutes time intervals up to 40 minutes. After each 10 minutes interval beads were recovered, dried and weighed in digital balance to determine its swelling property [20].

#### *In vitro* release kinetics

*In vitro* release behavior of ASVS from alginate encapsulated beads was studied kinetically according to Li et al. [19] with minute modification. An amount of 10 mg of alginate entrapped ASVS beads were taken in 10 ml of HCl- KCl buffer (pH-1.2), PBS (pH- 6.8), PBS (pH-7.0), and PBS (pH-7.4) and were incubated at 37°C on a shaker for 4 h. 1000 µl of buffer was taken out from all the mixture after every 30 minutes time interval and ASVS concentration was measured as protein concentration by absorption in spectrophotometer at 280 nm and 260 nm wavelengths with a correction factor:
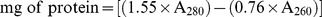
After measurement the fluid was returned back to the solution from where it was taken out [20].

#### Mucoadhesion study

Mucin binding study was performed depending upon the principle of reaction between periodic acid Schiff (PAS) and mucin [21], according to Dhawan et al. [22]. In brief, 10 mg of alginate entrapped ASVS beads were incubated in 2 ml of mucin solution (1 mg/ml) for 1 h. After the incubation 0.2 ml of periodic acid was added into it and was again incubated for 2 h. 0.2 ml of Schiff reagent was then added into it and incubated for 30 minutes at room temperature. After 30 minutes alginate entrapped ASVS beads were pelleted out by centrifuging the solution at 2000 rpm for 10 minutes and the absorbance of the supernatant was measured at 555 nm in spectrophotometer. The amount of mucin in the supernatant was determined from the standard curve of mucin solution (0.2 to 1 mg/ml) [22].

*Ex vivo* mucoadhesion study was performed according to Shelma and Sharma [23], with minute modification using freshly excised rat intestinal mucosa. Excised jejunum portion of rat intestine was flushed with physiological saline to remove luminal contents. Around 10 cm intestinal tissue was cut opened and placed on a polyethelene support with the help of cyanoacrylate adhesive. 100 particle of alginate beads as well as alginate entrapped ASVS beads were uniformly spread over it and allowed to interact with mucosa gel layer for 5 minutes. Tissues were then mounted on a platform at an angle of 45° and washed with phosphate buffer saline (pH 7.4) under a constant flow (10 ml/minutes). After 20 minutes, the particles which were attached to the intestine were quantified.

### Characterization of ASVS and released ASVS

ASVS solution was prepared using physiological saline and protein concentration was measured [18]. 1 gm of ASVS: alginate:: 1∶1 beads were taken in a beaker with 4 ml of phosphate buffer solution (10 mM, pH 7.0) and kept for 4 h to complete the release of encapsulated ASVS. Released protein was collected and concentration was measured [18]. Native polyacrelamide gel electrophoresis (12.5%) (PAGE) was performed to observe the banding pattern of normal ASVS and released ASVS after coomassie brilliant blue staining. High performance liquid chromatography of ASVS and released ASVS were performed using C18 column (4 mm×250 mm, flow rate: 0.5 ml/minutes, solvent: 60∶40∶0.2:: methanol: water: acetic acid).

### *In vitro* simulation of acid digestion in stomach

Acid digestion study was performed according to Li et al. [19]. 20 mg of alginate entrapped ASVS beads were taken in a 10 ml beaker. 0.5 ml of hydrochloric acid (0.01M) was added into it and was placed in 37°C for 2 h. After 2 h reaction was stopped and neutralized by addition of 0.01M sodium hydroxide solution. After neutralization volume of the solution was made up to 4 ml with phosphate buffer (pH 7.0) and placed in 37°C for 24 h for complete release of ASVS. It was then centrifuged and ASVS concentration of the supernatant was measured as protein concentration according to ultraviolet absorption. The activity of the released ASVS after digestion was assessed by phospholipase A_2_ (PLA_2_) inhibition assay.

### *Ex vivo* simulation of intestinal absorption

To study ASVS absorption ASVS was tagged with FITC. Overnight dialysis was performed to washout unbound FITC from the solution. This FITC tagged ASVS was then used in isolated intestinal preparation for absorption kinetic study. At first jejunum section of intestine (about 10 cm) was isolated from male Swiss albino mice under phenobarbital anesthesia and washed with Krebs-Ringer bicarbonate solution, pH 7.4. One side of the intestine was tied and FITC tagged ASVS in Krebs-Ringer bicarbonate solution was poured into the intestine through the hypodermic needle and then other side was also tied. The intestine was placed in a medium standard with 95% O_2_, 5% CO_2_ in phosphate buffer solution pH 7.4 at 37°C. O_2_ and CO_2_ mixture was bubbled into the solution to obtain intestinal peristaltic movement. After every one hour interval, 50 µl of buffer samples from the medium outside the intestine were collected and FITC intensity of that solution was measured using spectroflurometer (Jasco FP-6200, Japan). Excitation at 490 nm was used for FITC tagged ASVS and 505 nm to 530 nm emission spectra was recorded. A bandwidth of 5 nm was used for the assay. At the end of the assay the intestinal tissue was washed to remove unabsorbed FITC into the intestinal layer and it was homogenized. Intensity of FITC in the supernatant from the homogenate was again analyzed in same measurement by spectroflurometer using the same excitation and emission wavelengths respectively, to indentify incorporation of ASVS within the intestinal layers.

ASVS absorbed from the *ex vivo* intestinal preparation was collected from the medium outside the intestine compartment of the preparation, concentrated and pre-incubated with venom for neutralization studies.

### Phospholipase A_2_ enzyme assay

Phospholipase A_2_ activity of *Naja naja* and *Daboia russelii* venom was measured according to Dole, 1956 [24]. In this method free fatty acids released from egg yolk phospholipids suspended in 1% triton ×100, 0.1 M Tris HCL, 0.01M CaCl_2_,pH-8.5 buffer was titrated against snake venom (5 µg). It was incubated at 37°C for 15 minutes. Results are depicted as inhibition percentage, where 100% is the activity induced by snake venom alone.

### Neutralization of hemolytic activity

*Naja naja* venom was taken for study of hemolytic activity. Blood was collected aseptically from left radial vein of male healthy volunteers in heparinised vial and centrifuged at 3000 rpm for 15 minutes. The supernatant was discarded and the pellet containing human red blood cells (HRBC) were washed thrice with normal physiological saline by repeated centrifugation at 3000 rpm for 15 minutes. An erythrocyte suspension (hematocrit of 20%) was prepared and 250 µl of it was taken in each tube to which 50 µl *Naja naja* venom of different doses (2.5 µg–40 µg) were added. All the tubes were incubated for 1 h at 37°C. After incubation RBC solution was centrifuged at 3000 rpm for 15 minutes. The supernatant was separated and hemoglobin concentration was measured spectrophotometrically at 540 nm. Median hemolytic dose was calculated and ∼60% of the median hemolytic dose (10 µg) was used for neutralization study with normal ASVS, ASVS released from alginate beads. In brief, different concentration of normal ASVS (2.5 µg–50 µg)/released and absorbed ASVS (2.5 µg–50 µg) was incubated with 10 µg of venom for 1 h at 37°C. After incubation it was centrifuged at 2000 rpm for 15 minutes and the supernatant was incubated with 20% RBC suspension (250 µl) for 1 h at 37°C. After incubation RBC solution was centrifuged at 3000 rpm for 15 minutes. The supernatant was separated and hemoglobin concentration was measured spectrophotometrically at 540 nm [25].

### Inhibition of LDH assay

Different concentration of ASVS (25 µg) and released ASVS (25 µg) was incubated with 10 µg of *Naja naja* venom for 1 h at 37°C. After incubation it was centrifuged and the supernatant was incubated with 20% RBC suspension (250 µl) for 1 h at 37°C. After incubation RBC solution was centrifuged at 3000 rpm for 15 minutes supernatant was separated and lactate dehydrogenase level of the supernatant was measured spectrophotometrically at 340 nm as indicated in the assay kit.

### Neutralization of hemorrhagic activity

Hemorrhagic activity was quantitatively determined by the method of Kondo et al. [26] using mice as described by Sânchez et al. [27]. Hemorrhagic activity was assessed by injecting 0.1 ml (10 µg) of *Daboia russelii* venom in saline intradermaly into the back of the mice (20±2 gm). The hemorrhagic activity was estimated by measuring the diameter (cm) on the visceral side after 24 hour of intradermal injection. Neutralization was performed by both normal ASVS and released ASVS at the same concentration (25 µg).

### Neutralization of minimum lethal dose

The lethality of *Daboia russelii* venom and *Naja naja* venom was assessed by injecting different concentration of venom in 0.2 ml of 0.9% physiological saline (pH 7.4) into tail vein of male albino mice (20±2 gm) [28] and survivality was recorded up to 24 h. After determining the minimum lethal dose (MLD), neutralization of MLD was studied with ASVS and absorbed ASVS. In brief, normal ASVS (25 µg)/released and absorbed ASVS (25 µg) were incubated with venom (MLD dose) for 1 h at 37°C and centrifuged at 2000 rpm for 10 minutes. The supernatant was injected into the tail vein and neutralization was assessed for 24 h.

### Thermo stability of alginate coated ASVS

Thermo stability of alginate coated ASVS was evaluated by keeping those protein loaded beads at room temperature for 30 days and then neutralization study was performed with the released protein from those beads as described above against russel viper venom.

### Oral delivery of alginate coated ASVS

#### Neutralization of minimum lethal dose

The lethality of *Daboia russelii* venom was assessed by intra muscular injection of different concentration of venom in 0.1 ml of 0.9% physiological saline (pH 7.4) in male albino rat (200±20 gm) [28] and survivality was recorded up to 24 h. After determining the minimum lethal dose (MLD), neutralization of MLD was studied with perorally treated alginate coated ASVS. In brief, after injecting the venom intramuscularly alginate coated ASVS bead (30 mg beads equivalent to 800 µg protein) was per orally introduced at 0^th^minute and every 15 minutes intervals up to 6 hours followed by 30 minutes interval up to 12 hours.

#### Neutralization of hemotoxicity

Morphological alteration of RBC was studied by collecting blood from retro orbital plexus after 2 hours of venom injections from venom injected animals and venom injected perorally alginate coated ASVS fed animals. Blood film was drawn, dried and RBC was subjected to morphological analysis by compound microscopy at 40×.

Clotting time was measured after Clemens et al. 1995 [29]. Intramuscular MLD dose of venom was administered and clotting time was monitored from retro orbital blood at a regular 30 minutes interval up to 4 hours. Same experiment was also repeated with venom injected alginate coated ASVS per orally fed animals.

Hemolysis study was also performed after 6 hours of venom injection from venom injected animals and from venom injected perorally alginate coated ASVS fed animals. In brief blood was collected, plasma was separated and plasma free hemoglobin concentration was measured by Drabkin's method using the following formulae
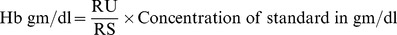
Spectrum was also recorded from 300 nm up to 750 nm using Simatzu spectrophotometer (model no:1800).

#### Venom induced renal toxicity study

Venom was injected intramuscularly at MLD dose and after 6 hours, venom injected animals and venom injected perorally alginate coated ASVS fed animals were sacrificed. Blood was collected and plasma was separated. Plasma urea and creatinine level was measured using respective assay kit following manufacturer's instructions.

After animals were sacrificed, kidney was excised and fixed in 10% buffered formalin for 24 hours. Tissues were dehydrated, processed, and embedded in paraffin wax (melting point: 56±2°C). Blocks were prepared and 5 µm thick sections from each block were prepared. Tissue sections were stained with haematoxylin and eosin, histopathological alterations were evaluated under compound microscope (10×).

### Statistical analysis

All the results were expressed as mean±SEM, n = 6. Level of significance was determined by one way ANOVA followed by Tukey's *post hoc* test. *p*<0.05 was considered as significant.

## Results

### Entrapment efficiency and loading capacity

Entrapment efficiency and loading capacity were studied by varying alginate and ASVS concentration in three different ratios (alginate: ASVS:: 2∶1, alginate: ASVS:: 1∶1, and alginate: ASVS:: 1∶2 in v/v) to determine optimum condition for maximum entrapment of ASVS into alginate. The entrapment efficiency was found to be 20.2±0.67% in alginate: ASVS:: 2∶1, 37.2±0.73% in alginate: ASVS:: 1∶1 and 33.7±0.99% in alginate: ASVS:: 1∶2 when chelated in 2% calcium chloride solution and 21.6±0.86% in alginate: ASVS:: 2∶1, 37.3±0.94% in alginate: ASVS:: 1∶1 and 35.0±0.71% in alginate: ASVS:: 1∶2 when chelated in 3% calcium chloride.

Entrapment efficiency was found to be in the order of alginate: ASVS:: 1∶1 beads>alginate: ASVS:: 1∶2 beads>alginate: ASVS:: 2∶1 in 2% as well as 3% in calcium chloride solution (Fig. 1D).

Loading capacity was found to be in the order of alginate: ASVS:: 1∶2 beads>alginate: ASVS:: 1∶1 beads>alginate: ASVS:: 2∶1 beads in 2% as well as in 3% calcium chloride solution (Fig. 1B). But as the entrapment efficiency is maximum in alginate: ASVS:: 1∶1 ratio, it was used for further study (Fig. 1E).

### Scanning electron microscopy study

The scanning electron microscopy study showed that beads were spherical and has more homogenous surface in alginate: ASVS:: 1∶1 beads as compared with alginate: ASVS:: 1∶2 beads (Fig. 1F, 1G, 1H). Respective surface analysis result confirmed that the surface heterogeneity was increased in alginate: ASVS:: 1∶2 beads as compared with alginate: ASVS:: 1∶1 beads (Fig. 1F′, 1G′, 1H′).

### Swelling study

**Figure 2:**
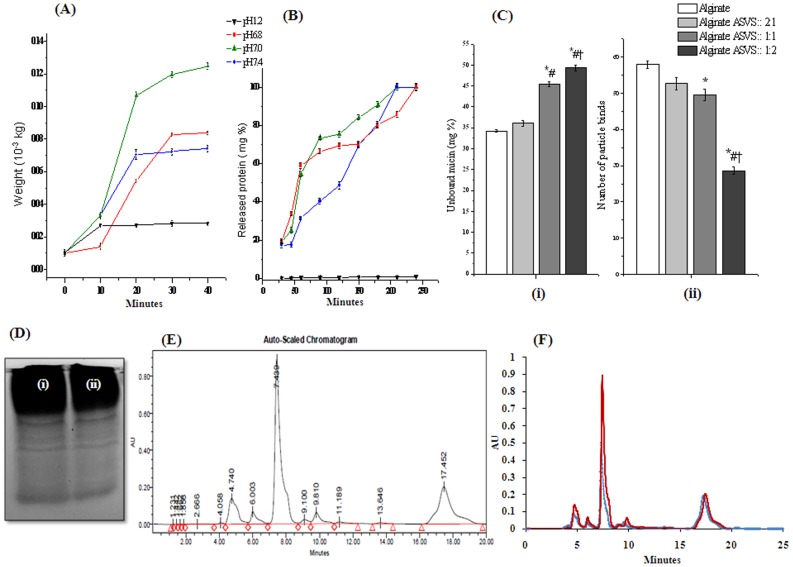
Swelling, *in-vitro* release, mucoadhesion capacity study of alginate-ASVS beads and characterization of normal, released ASVS. (A) Swelling property of alginate entrapped ASVS beads (alginate: ASVS :: 1∶1) at different pH solution. (B) Release profile of alginate entrapped ASVS beads (alginate: ASVS :: 1∶1) at different pH solution. Result showed as mean ± SEM, n = 6. (Ci) Mucin binding study of alginate entrapped ASVS beads at different concentration ratio of alginate: ASVS :: 2∶1; alginate: ASVS :: 1∶1; alginate: ASVS :: 1∶2 in 2% calcium chloride solution. (Cii) *Ex vivo* mucoadhesion study of alginate entrapped ASVS beads at different concentration ratio of alginate: ASVS :: 2∶1; alginate: ASVS :: 1∶1; alginate: ASVS :: 1∶2 in 2% calcium chloride solution. Result showed as mean ±SEM, n = 6. *# † *p*<0.05 was considered significant (*Alginate vs alginate: ASVS :: 2∶1, alginate: ASVS :: 1∶1, and alginate: ASVS :: 1∶2; # Alginate: ASVS :: 2∶1 vs alginate: ASVS :: 1∶1, alginate: ASVS :: 1∶2; † Alginate: ASVS :: 1∶1 vs alginate: ASVS :: 1∶2). (Di) Native polyacrelamide gel electrophoresis of normal ASVS, (Dii) Native polyacrelamide gel electrophoresis of released ASVS. (E) HPLC of normal ASVS solution (C18 column, 4 mm×250 mm, flow rate: 0.5 ml/min, solvent: 60∶40∶0.2:: methanol: water: acetic acid); (F) Overlay of HPLC data of normal ASVS solution and released ASVS solution from alginate beads.

The swelling ability of alginate entrapped ASVS beads were studied using different pH solution of 1.2, 6.8, 7.0 and 7.4. Alginate entrapped ASVS beads did not show any significant swelling at the pH 1.2 after 40 minutes of incubation whereas it showed significant gradual increase in swelling after 10, 20, 30 and 40 minutes of incubation respectively at pH 6.8, 7.0 and 7.4 solution which was expressed as weight change of beads, shown in Fig. 2A. Weight of alginate entrapped ASVS beads were increased by 1.4, 3.3, 3.3 times at pH 6.8, 7.0, 7.4 after 10 minutes incubation, Weight of alginate entrapped ASVS beads were increased by 5.4, 9.2, 7.04 times at pH 6.8, 7.0, 7.4 after 20 min incubation, by 8.2, 10.62, 7.24 times at pH 6.8, 7.0, 7.4 after 30 minutes incubation, and by 8.4, 12.48, 7.4 times at pH 6.8, 7.0, 7.4 after 40 minutes incubation respectively.

### *In vitro* release study

*In vitro* release of ASVS from alginate entrapped beads was studied in different pH solution of 1.2, 6.8, 7.0, and 7.4, simulating gastrointestinal conditions for 4 h (Fig. 2B). From the figure it was found that burst release of ASVS took place after 1 h of incubation at 6.8 and 7.0 pH solution respectively which then gradually reached its maximum capacity. At pH 7.4 solution alginate entrapped beads showed a steady and firm release of ASVS which continuously increased from 1 h and reached to the maximum at 4 h. The release of ASVS from alginate entrapped beads at pH 1.2 was found to be significantly lower from pH 6.8, 7.0 and 7.4 solution.

### Mucoadhesion study by mucin binding study and *ex vivo* mucoadhesion study

Mucus glycoprotein assay was performed and the result was depicted in Fig. 2Ci. Result was calculated by measuring the unbound mucin concentration spectrophotometrically at 555 nm. Concentration of the free mucin in the solution were 34.22% for alginate beads, 36.04% alginate: ASVS:: 2∶1 beads, 45.49% for alginate: ASVS:: 1∶1 beads and 49.33% for alginate: ASVS:: 1∶2 beads. Thus, from the above result it was found that mucoadhesive property of alginate is altered with the amount of ASVS entrapped. Maximum free mucin was found in the condition where alginate: ASVS:: 1∶2 beads were incubated with mucin solution and lowest free mucin was found in case of pure alginate beads.

*Ex vivo* mucoadhesion study was performed using rat intestine. Results showed that particle binding capacity of alginate: ASVS:: 2∶1 beads were 91.07%, alginate: ASVS:: 1∶1 beads were 85.6% and alginate: ASVS:: 1∶2 beads were 49.56%, when binding capacity of pure alginate beads were consider as 100%. Thus, maximum particle binding was observed in alginate: ASVS:: 2∶1 beads which was not significantly different from pure alginate beads and alginate: ASVS:: 1∶1 beads and with increase in ASVS concentration particle binding capacity significantly decreased in alginate: ASVS:: 1∶2 beads (Fig. 2Cii).

### Characterization of ASVS and released ASVS

Normal ASVS and released ASVS showed presence of similar banding patterns after coomassie brilliant blue staining in native PAGE (Fig. 2Di, 2Dii). Result of HPLC showed the presence of multiple peaks in normal ASVS solution (Fig. 2E). Similar HPLC peak pattern of released ASVS (2F) indicated the presence of all protein fractions in released ASVS solution.

### Acid digestion

**Figure 3:**
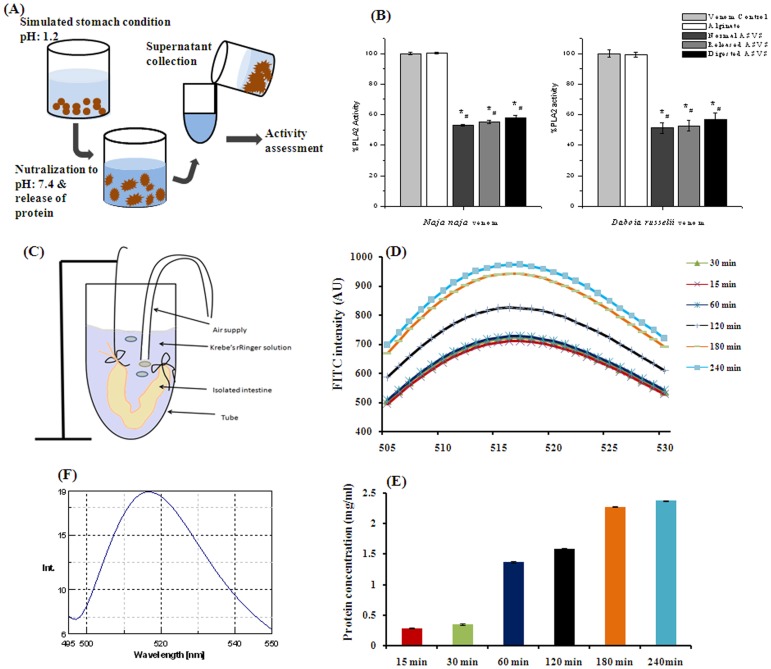
Venom activity neutralization by ASVS after acid digestion and intestinal absorption in simulated gut conditions. (A) Schematic diagram of *ex vivo* simulation of acid digestion in stomach; (B) *In vitro Naja naja* venom and *Daboia russelii* venom phospholipase A_2_ inhibitory activity of normal ASVS, released ASVS which was not acid digested and acid digested released ASVS. Result showed as mean ±SEM, n = 6. *# *p*<0.05 was considered significant (* Venom control vs normal ASVS, released ASVS, and digested ASVS, # Alginate vs normal ASVS, released ASVS, and digested ASVS). (C) Schematic diagram of *ex vivo* simulation of intestinal absorption; (D) Pharmacokinetics of FITC tagged ASVS absorption from outer intestinal fluid by monitoring FITC intensity in spectroflurometer; (E) Monitoring of ASVS concentration in outer intestinal fluid for 4 h; (F) Monitoring of ASVS from FITC intensity within the intestinal layer of mice after 4 h.

Acid digestion of particle was made by placing alginate: ASVS:: 1∶1 beads in 0.1M HCl for 1 h and then the encapsulated ASVS was recovered by placing the beads in phosphate buffer (pH 7.0) for assessing its activity. In the activity study it was found that the released ASVS from alginate: ASVS:: 1∶1 acid digested beads showed significant protection by 41.09% against PLA_2_ enzyme activity of *Naja naja* venom and by 43.16% against PLA_2_ enzyme activity of *Daboia russelii* venom which was not significantly different in released ASVS from alginate: ASVS:: 1∶1 beads which was not acid digested. This data indicates that alginate entrapment might protect ASVS to overcome the damage caused by the acidic environment of the gastrointestinal tract (Fig. 3A, 3B).

### Neutralization of venom by ASVS after intestinal absorption in simulated gut conditions

Presence of FITC emission at 520 nm after excitation at 490 nm of the fluid from outer intestinal medium indicated that FITC tagged ASVS was permeated across the intestinal barrier. A time dependent gradual increase in FITC tagged ASVS concentration was observed up to 4 hour which was started from 15^th^ minutes (Fig. 3C, 3D, 3E). The permeated concentrated ASVS also showed neutralization of *Daboia russelii* venom and *Naja naja* venom induced lethality in male albino mice.

Emission of FITC from the intestinal tissue homogenate in the same experimental condition also indicated the transport of FITC tagged ASVS through the intestine (Fig. 3F).

### Phospholipase A_2_enzyme assay

The PLA_2_ enzyme activity study was performed to find out the activity of released ASVS from alginate: ASVS:: 1∶1 beads as well as normal ASVS. In the present experiment it was found that ASVS showed a significant 47.88%, 48%and79% protection against PLA_2_ enzyme activity of *Naja naja* venom and *Daboia russelii* venom whereas the released ASVS from alginate: ASVS:: 1∶1 beads showed a significant 45.79% and 47.35% protection against PLA_2_ enzyme activity of *Naja naja* venom and *Daboia russelii* venom respectively as shown in Fig. 3B.

### *In vitro* hemolytic activity

**Figure 4:**
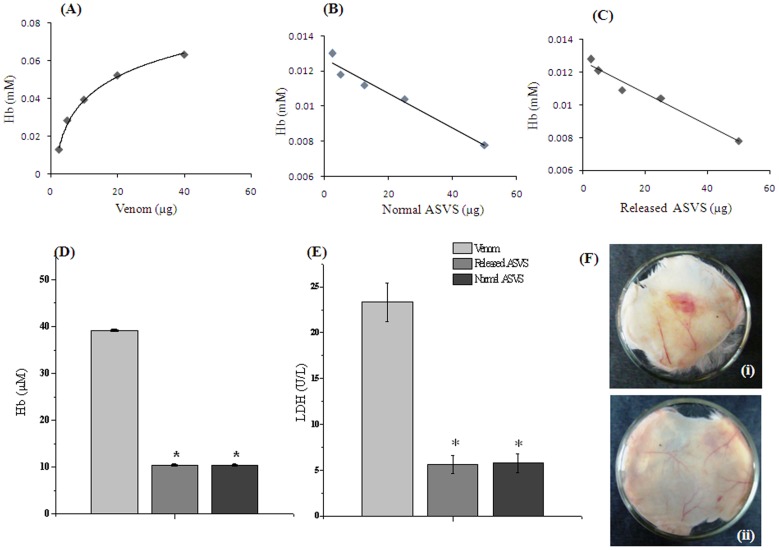
Neutralization of hemolysis activity and hemorrhagic activity of venom by normal ASVS and released ASVS. (A) Dose dependent hemolytic activity of *Naja naja* venom on HRBC (B): Dose dependent protective efficacy of normal ASVS against *Naja naja* venom induced hemolysis. (C): Dose dependent protective efficacy of released ASVS against *Naja naja* venom induced hemolysis. (D): Comparison of anti-hemolytic activity of normal ASVS and released ASVS on *Naja naja* venom induced hemolysis. (E) Effect of normal ASVS and released ASVS on LDH level of HRBC hemolysate. Result showed as mean ±SEM, n = 6. *p<0.05 was considered significant. * Venom control vs normal ASVS, released ASVS. (Fi) Hemorrhagic activity of *Daboia russelii* venom; (Fii) Neutralization of hemorrhagic activity of *Daboia russelii* venom by released ASVS from alginate beads.

*In vitro* hemolysis of RBC was increased dose dependently after *Naja naja* venom incubation (2.5 µg–40 µg) as was observed from hemoglobin concentration of sample supernatant. The median hemolytic dose of *Naja naja* venom was found to be 17.45 µg in 20% RBC solution (Fig. 4A). Released ASVS dose dependently (2.5 µg–50 µg) neutralized the hemolytic activity of *Naja naja* venom (10 µg) significantly. The median neutralization dose of normal ASVS and released ASVS hemolytic activity of *Naja naja* venom was found to be 12.5 µg and 12.05 µg respectively (Fig. 4B, 4C). The neutralization capacity of hemolytic activity for released ASVS did not show any significant alteration as compared with normal ASVS in a similar 25 µg dose (Fig. 4D).

### Lactate dehydrogenase assay

Lactate dehydrogenase concentration of RBC hemolysate significantly increased after incubation with 10 µg of *Naja naja* venom. Normal ASVS, and released ASVS treatment significantly decreased lactate dehydrogenase concentration of RBC hemolysate after incubation with 10 µg of *Naja naja* venom. No significant difference was observed in lactate dehydrogenase level of HRBC hemolysate in normal ASVS, and released ASVS treatment (Fig. 4E).

### Neutralization of hemorrhagic activity

Normal and released ASVS (25 µg) showed to neutralize hemorrhagic activity of *Daboia russelii* venom (10 µg) significantly (Fig. 4F).

### Neutralization of MLD

Minimum lethal dose (MLD) of *Naja naja* venom was found to be 6 µg/20 gm mice while that of *Daboia russelii* venom was found to be 4 µg/20 gm mice. These respective MLDs of *Naja naja* and *Daboia russelii* venoms were significantly neutralized by 24.108 µg of released ASVS as well as similar amount of normal ASVS.

### Thermo stability of alginate coated ASVS

The minimum lethal dose of *Daboia russelii* venom was found to be 4 µg/20 gm mice. Protein released from the alginate coated ASVS beads after 30 days incubation at 37°C showed a complete neutralization of lethality at a dose of 26 µg protein which did not show any significant difference from normal ASVS.

### Oral delivery of alginate coated ASVS

#### Neutralization of minimum lethal dose

Minimum lethal dose (MLD) of *Daboia russelii* venom was found to be 1 mg/kg intramuscularly in white albino Wistar strain rat. Oral delivery of 30 mg beads which contains approximately 800 µg protein in regular 15 minutes interval up to 6 hours and 30 minutes interval up to 12 hours showed to neutralize MLD of *Daboia russelii* venom in treated group of rats.

#### Hemotoxicity study

**Figure 5:**
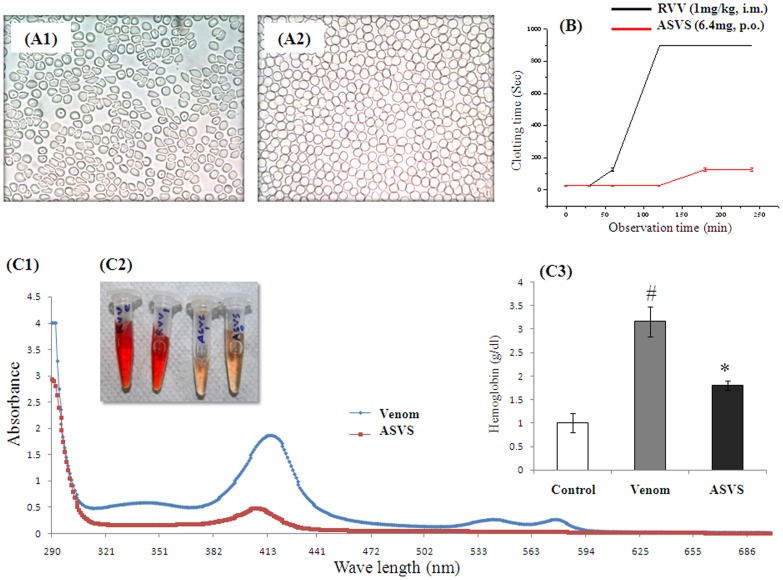
*In vivo* neutralization of venom induced hemotoxicity. (A1) RBC morphological alteration after venom induction (A2) RBC morphological alteration in alginate coated ASVS fed animals after venom induction, (B) Venom induced clotting time alterations, (C) Hemolysis assay. (C1) Spectrum of plasma hemoglobin, (C2) Pictorial depiction of plasma of venom injected animal and venom injected perorally alginate coated ASVS fed animal. (C3) Assessment of plasma free hemoglobin concentration measurement by Drabkin's methods. Result showed as mean ±SEM, n = 6. *p<0.05 was considered significant. # Normal control animals vs Venom control animal, * Venom control animal vs alginate coated ASVS fed animal.

Intramuscular administration of *Daboia russelii* venom (1 mg/kg) significantly alter erythrocyte morphology after 120 minutes as seen in light microscopy. Normal discocytic cells have been altered to different morphological shapes like spherocytes, echinocytes, stomatocytes, etc., thereby decreasing the population of discocytes. Oral ASVS beads treatment found to prevent this morphological alterations (Fig. 5A, 5B) in treated group of animals.

Intramuscular administration of *Daboia russelii* venom (1 mg/kg) significantly increased clotting time after 120 minutes compared to normal animals. Figure 5B showed that the clotting time reaches greater than15 minutes from the 120 minutes after venom injection. Oral delivery of alginate coated ASVS beads showed delay in increase in clotting time in treated group of animals as compared with venom injected group of animals (Fig. 5B)

Plasma free hemoglobin concentration was increased in venom injected group of animals as compared with normal group of animals. Oral delivery of 30 mg alginate coated ASVS beads (800 µg) in every 15 minutes up to 6 hours showed lower level of free hemoglobin in plasma by 43.16% in treated group of animals as compared with venom injected group of animals (Fig. 5C1,C2,C3).

#### Venom induced renal toxicity study

**Figure 6:**
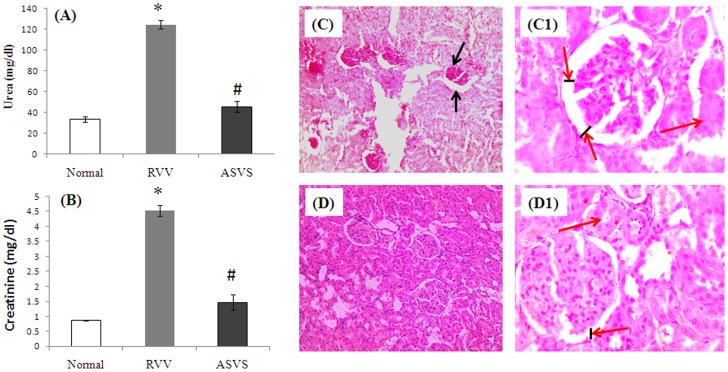
*In vivo* neutralization of venom induced renal toxicity. (A) Measurement of plasma urea concentration in both venom induced group as well as alginate coated ASVS fed animals. (B) Measurement of plasma creatinine concentration in both venom induced group as well as alginate coated ASVS fed animals. (C) Microscopic image of venom induced alteration in renal tissues after hematoxylin and eosin staining at 10×. (C1) Microscopic image of venom induced alteration in renal tissues after hematoxylin and eosin staining at 40×. (D and D1) Microscopic image of renal tissue of alginate coated ASVS bead fed animals after hematoxylin and eosin staining at 10× and 40× respectively.

After 6 hours of intramuscular administration of *Daboia russelii* venom (1 mg/kg) plasma creatinine and urea level significantly elevated in venom injected animals as compared with normal group of rats. Oral delivery of 30 mg alginate coated ASVS beads in every 30 minutes up to 6 hour showed significantly decreased level of plasma urea and creatinine by 63.31% and 67.55% in treated group of animals as compared with venom injected group of animals (Fig. 6A, 6B).

Histopathological studies revealed alteration in glomerulous as well as in tubular part of kidney after eosin and hematoxylin staining after venom injection. Glomerular membranes undergo degeneration as well as acute tubular necrotic features in venom injected animals which were restored in orally alginate coated ASVS bead treated group of animals (Fig. 6C, 6C1, 6D, 6D1).

## Discussion

### Choice of delivery system

The aim of present study was to entrap multiple proteins of ASVS in alginate beads which will be functionally active against snake venom upon release. So far oral delivery systems have dealt with simple and small proteins like insulin or BSA [9], [30] or vaccine, which use different strategy for delivery system [31]. From drug delivery point ASVS has some peculiarities which make it worth individual attention. The ASVS comprises of immunoglobulins raised biologically in animals against venom. The ASVS which was used in this study provided antibody against four major Indian snake venoms (cobra, common krait, russell viper, saw scaled viper). ASVS contains multiple proteins which vary and thus, the chemical properties, isoelectric point or neutralization of venom components by individual proteins has not been studied in detail. Moreover, as ASVS is not a lucrative business [32], making sophisticated oral delivery system is a commercially unviable project. To overcome these problems the different protein components were not given individual consideration; rather those were encapsulated as a whole in alginate, a cheap polymer, cross linked by divalent cation calcium. This delivery system has the capacity to entrap proteins with different isoelectric pHs unlike ionic gelation method [33] and therefore may proof efficient for entrapping every component of the multiple components of ASVS. Results showed (Fig. 2D, 2E, 2F) that this system indeed was proved efficient in entrapping every component of the multiple component ASVS.

The influence of preparation condition like the concentration of the polymer alginate as well as the concentration of calcium chloride solution will be different for encapsulation of different proteins [34], [35]. In the present study alginate: ASVS:: 1∶1 beads were used as it showed maximum entrapment efficiency though the loading capacity was higher at alginate: ASVS:: 1∶2. The SEM study too confers less heterogeneity of alginate: ASVS:: 1∶1 beads. The calcium chloride concentration (2% or 3%) variation did not show significant alteration in entrapment efficiency or loading capacity. Therefore 2% calcium chloride solution was used for the preparation of beads for the biological studies.

pH dependent swelling property is one of the advantageous phenomenon of alginate beads, widely used in oral drug delivery. The alginate meshwork swells or shrinks respectively in alkaline and acidic pH, which helps to release or entrap proteins within alginate beads [36].Shrinking the beads in low pH ensure protection of entrapped protein from digestion within stomach. Result showed that at pH 1.2 the alginate beads did not swell but with increase in pH the swelling property increases. At pH 7.0 the beads swell maximally, ensuring release of entrapped protein in the intestine.

*In vitro* release of protein from polymer was studied in different pH, simulating gastrointestinal condition. The release pattern of ASVS from alginate beads showed an initial burst release in alkaline pH. Though it is considered an undesirable and uncontrollable phenomenon as it prevent sustain release and creates problem in many controlled delivery systems but burst release is beneficial in situations like wound healing [37]. An initial burst is necessary to provide immediate relief, followed by sustained release to maintain the action, as is the situation in snake envenomation, similar to wound healing, the burst release may be beneficial.

One of the main purposes of the study was to encapsulate all the protein components of ASVS within polymer and released them in the intestinal environment so that the polyvalent nature of ASVS and its bioactivity could be maintained. Similar banding pattern in native PAGE and similar chromatogram pattern in HPLC of released ASVS in comparison with normal ASVS indicated that all the components of normal ASVS were present in released ASVS. Thus, these results confirm the entrapment of every protein component of ASVS into alginate beads.

The released proteins are susceptible to intestinal protease activity. Mucoadhesive drug delivery systems increase the bioavailability of drug by increasing the contact and residence time of the drug on gastrointestinal tract, the absorption surface. Mucoadhesive polymers can bind to mucus by physical or chemical interactions. Alginate, consisting of mannuronic acid and guluronic acid forms, has more hydroxyl groups than the other polymers and thereby binds more strongly with the oligosaccharide chains of mucin [38]. Mucoadhesive property of alginate and alginate encapsulated ASVS of different concentrations were studied in two different models. Despite the difference between alginate beads and ASVS loaded alginate beads in mucin binding property, ASVS encapsulated alginate beads adhere to rat gastric mucosa significantly. So it could be assumed that alginate encapsulated ASVS beads could be able to bind with gastric mucosa in significant amount.

### Venom neutralization potential

After characterization of the bead it was necessary to study whether released ASVS could permeate the intestinal barrier. An isolated intestinal preparation was used to study this phenomenon. The flurochrome FITC was tagged with ASVS and after excess unbound FITC was washed out, the permeability was assessed by measuring FITC intensity kinetically from the outer intestinal fluid. This is an alternative model of *in vivo* absorption study where it was found that FITC intensity was started to increase from 15 minutes of the experiment gradually up to 4 hours. FITC tagged ASVS concentration was also increased gradually from 15 minutes to 4 hours. FITC tagged ASVS from outer intestinal fluid significantly neutralized the venom induced lethality which signify that all the major components required for venom induced lethality neutralization was absorbed through the intestine.

Further experiments with the released proteins from beads were performed to study whether any alteration in biological functions of the released ASVS had occurred due to entrapment or during release from the beads as compared with normal ASVS. The released protein was thus used to evaluate the functional aspects of ASVS. WHO [39] has recommended assay of ASVS based on neutralization of lethality in experimental animals. In the present study this assay was performed using two different kinds of venom viz *Naja naja* and *Daboia russelii*. It was found that the released ASVS neutralizes the lethality of both venoms. But neutralization of lethality does not necessarily correlate with neutralization of specific venom actions [40] and death can occur due to these actions beyond the time frame taken for lethality studies. For this reason *in vitro* neutralization of the PLA_2_ activity; [41], [42], passive agglutination [43], hemolytic activity [44], hemorrhagic activity and ELISA [28], [45] have been substituting the *in vivo* method.

In the present study phospholipase A_2_ enzyme activity was measured to study the functional ability of the released ASVS protein in neutralizing the snake venom. Snake venom possesses PLA_2_ enzyme activity, which manifests many of the toxic effects of the venom [46]. In this study released ASVS showed significant inhibition of venom PLA_2_ activity. Naja venom possesses direct hemolytic factor [27] which dose dependently showed hemolysis in washed RBC solution. Released ASVS also showed significant protection against hemolytic activity dose dependently. Lactate dehydrogenase is a marker for cellular toxicity and hemolysis. Released ASVS also significantly restored LDH released from RBC lysate. These activities of released ASVS were comparable with freshly constructed ASVS which clearly indicate that the biological activity of ASVS remains unaltered after entrapment and also after release from the alginate beads. A similar result was obtained from hemorrhagic activity study with viper venom. Intradermal injection of viper venom causes local manifestations such as hemorrhage which was also neutralized by released ASVS.

**Figure 7:**
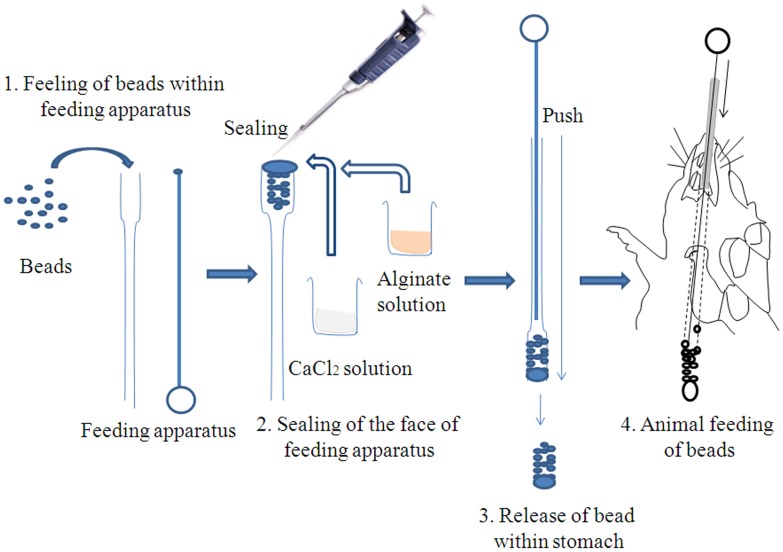
Schematic diagram of oral delivery procedure of alginate coated ASVS beads.

The gold standard to test ASVS activity is neutralization of venom lethality in animals done by injecting preincubated venom and antivenom in experimental animals. Since oral formulation cannot be pre-incubated we have shown lethality neutralization of ASVS released from alginate bead after crossing the intestinal barriers. This mimics the real situation as empirically as preincubated models and needs new methodology to study efficacy of ASVS as well as oral ASVS (Fig. 7). But the above studies cannot confirm that how much this oral delivery is beneficial as there are lots of other factors involved in it like peristaltic movement, effects of protein digestion enzymes as well as the uptake kinetics. Thus the feasibility of alginate coated ASVS bead to provide sufficient amount of antivenom required for neutralization of venom was studied in animal model. Alginate coated ASVS was given orally by the method of Matsuno et al 2008 [47] with minute modification (Fig. 7). Viper venom induced lethality, hemotoxicity and renal toxicity was chosen as venom toxicity models as these effects are irreversible and are affected by delay in ASVS administration. Alginate coated ASVS was per orally administered in every 30 minutes interval up to 6 hours for hemotoxic and nephrotoxic models and till 24 h for lethal model. Peroral administration of ASVS significantly prevented venom induced toxicity which is depicted by delay in increase of clotting time, inhibition of morphology alteration of RBC, decrease release of free hemoglobin, and low plasma urea and creatinine level as compared with venom induced group of animals. Above all neutralization of MLD has been found after peroral delivery of alginate coated ASVS. From these studies it has been found that oral delivery of alginate coated ASVS prevented the damage caused due to venom administration. The above study confers that oral delivery of ASVS could be possible and it could help to decrease number of deaths occurring due to snake bite in remote places.

Intestinal absorption does not delay ASVS availability in blood in a dose sufficient to neutralize venom infiltrated from muscle site before toxic effects of venom could be recognized. Being immunoglobulin in nature might have helped intestinal absorption of ASVS by direct binding with IgG-Fc receptor (FcRn) mediated transport, which can account for timely action of oral ASVS in venom neutralization in animal models.

### Observation on limitations of methodology

In this study we have selected alginate for its low cost. However, ASVS is expensive and the low loading capacity of alginate system has to be improved for better economy of ASVS. Alginate- antivenom bead will not reduce the cost of antivenom production. But, the increment in price of this novel antivenom when compared to the traditional one would not be much. Another limitation of this study which has to be detailed is the thermostability of alginate-ASVS preparation at room temperature. We have not studied whether the thermal stability of alginate-antivenom would be greater than that of the antivenom alone and beyond one month. This would imply that the alginate-antivenom mixture could not be stored at room temperature and would require cold chain conditions unless the polymer formulation is developed to address this problem. Approaches like addition of the polyols [48] to increase thermostability of liquid ASVS can address both the problems of cost and thermostability of freeze dried ASVS, and hence, such approaches have to be taken for improving our formulation. Moreover, oral ASVS can be given only as first aid and the minimum dose to prevent the irreversible damages taking place during transit. This warrants detail study with ASVS dosing separately for venom toxicities for choosing optimum dose and release kinetics as well as for pharmacokinetic and pharmacodynamic analysis.

### Conclusion and future prospect

**Figure 8:**
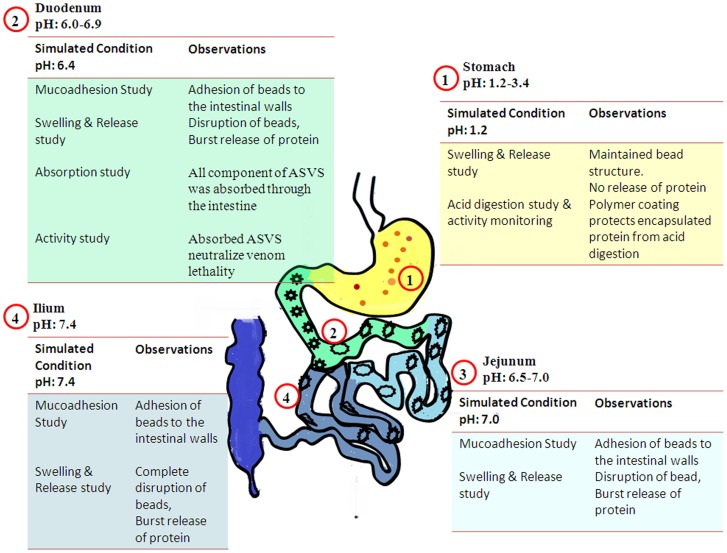
Schematic diagram of the mode of action of alginate coated ASVS beads after peroral delivery.

Antisnake venom serum is the only drug available against snake envenomation but is limited by infrastructure required to administer this intravenously as soon as the bite occurs. In this paper we have addressed this limitation by testing the feasibility of controlled oral delivery of the drug (Fig. 8) for future use as first aid. Advantages of alginate system were retained after ASVS loading and venom neutralizing properties of ASVS was unaltered after release from the beads. Hence, alginate could be used to develop controlled oral delivery system of ASVS but requires polymer modification for animal experiments and clinical trials. Moreover, this is the first report of encapsulating multiple proteins of a drug to reconstitute a functional drug, for which new strategies should be proposed.

## References

[1] WHO (2009) Neglected tropical diseases: snakebite. Available: http://www.who.int/neglected_diseases/diseases/snakebites/en/index.html Accessed 12 August 2009.

[2] Williams D, Gutiérrez JM, Harrison R, Warrell DA, White J, et al (2010) The Global Snake Bite Initiative: an antidote for snake bite. Lancet 375: 89–91. 20109867 10.1016/S0140-6736(09)61159-4

[3] Mohapatra B, Warrell DA, Suraweera W, Bhatia P, Dhingra N, et al (2011) Snakebite mortality in India: a nationally representative mortality survey. PLoS Negl Trop Dis 5 4: e1018 10.1371/journal.pntd.0001018 21532748 PMC3075236

[4] Brown NI (2012) Consequences of neglect: analysis of the sub-Saharan African snake antivenom market and the global context. PLoS Negl Trop Dis 6 6: e1670 10.1371/journal.pntd.0001670 22679521 PMC3367979

[5] Warrell DA (2010) Snake bite. Lancet 375: 77–88. 20109866 10.1016/S0140-6736(09)61754-2

[6] Alirol E, Sharma SK, Bawaskar HS, Kuch U, Chappuis F (2010) Snake bite in South Asia: a review. PLoS Negl Trop Dis 4 1: e603 10.1371/journal.pntd.0000603 20126271 PMC2811174

[7] Gutiérrez JM, Warrell DA, Williams DJ, Jensen S, Brown N, et al (2013) The need for full integration of snakebite envenoming within a global strategy to combat the neglected tropical diseases: the way forward. PLoS Negl Trop Dis 7 6: e2162 10.1371/journal.pntd.0002162 23785526 PMC3681653

[8] Hansson E, Sasa M, Mattisson K, Robles A, Gutiérrez JM (2013) Using geographical information systems to identify populations in need of improved accessibility to antivenom treatment for snakebite envenoming in Costa Rica. PLoS Negl Trop Dis 7 1: e2009 10.1371/journal.pntd.0002009 23383352 PMC3561131

[9] Suksamran T, Opanasopit P, Rojanarata T, NgawhirunpatT, Ruktanonchai U, et al (2009) Biodegradable alginate microparticles developed by electrohydrodynamic spraying techniques for oral delivery of protein. J Microencapsul 26: 563–570. 19839791 10.3109/02652040802500622

[10] Gaucher G, Satturwar P, Jones MC, Furtos A, Leroux JC (2010) Polymeric micelles for oral drug delivery. Eur J Pharm Biopharm 76: 147–158. 20600891 10.1016/j.ejpb.2010.06.007

[11] Bagan J, Paderni C, Termine N, Lo Russo L, Compilato D, et al (2012) Mucoadhesive Polymers for Oral Transmucosal Drug Delivery: A Review. Curr Pharm Des 18: 54 97–514. 10.2174/138161212803307545 22632395

[12] Maurer N, Fenske DB, Cullis PR (2001) Developments in liposomal drug delivery systems. Expert Opin Biol Ther 1: 923–947. 11728226 10.1517/14712598.1.6.923

[13] Park JW (2002) Liposome-based drug delivery in breast cancer treatment. Breast Cancer Res 4: 95–99. 12052251 10.1186/bcr432 PMC138729

[14] Zhao Y, Trewyn BG, Slowing II, Lin VSY (2009) Mesoporous Silica Nanoparticle-Based Double Drug Delivery System for Glucose-Responsive Controlled Release of Insulin and Cyclic AMP. Colloids and Surf B: Biointerfaces J Am Chem Soc 131: 8398–8400. 10.1021/ja901831u 19476380

[15] Singh R, Lillard JW Jr (2009) Nanoparticle-based targeted drug delivery. Exp Mol Pathol 86: 215–223. 19186176 10.1016/j.yexmp.2008.12.004 PMC3249419

[16] Jain KK (2012) Nanobiotechnology-based strategies for crossing the blood-brain barrier. Nanomedicine (Lond) 7: 1225–33. 22931448 10.2217/nnm.12.86

[17] Sarkar K, Srivastava R, Chatterjee UC, Kundu PP (2011) Evaluation of Chitosan and their self-assembled nanoparticles with pDNA for the application in gene therapy. JAPS 121: 2239–2249.

[18] Lowry OH, Rosebrough NT, Farr AL, Randall RJ (1951) Protein measurement with the Folinphenol reagent. J Biol Chem 193: 265–275. 14907713

[19] Li XY, Kong XY, Shi S, Zheng XL, Guo G, et al (2008) Preparation of alginate coated chitosan microparticles for vaccine delivery. BMC Biotechnol 8: 89. 19019229 10.1186/1472-6750-8-89 PMC2603011

[20] Shi XW, Du YM, Sun LP, Yang JH, Wang XH, et al (2005) Ionically crosslinked alginate/carboxymethyl chitin beads for oral delivery of protein drugs. Macromol Biosci 16: 881–889. 10.1002/mabi.200500063 16134086

[21] Mantle M, Allen AA (1978) Colorimetric assay for glycoproteins based on the periodic acid/Schiff stain [proceedings]. Biochem Soc Trans 6: 607–609. 208893 10.1042/bst0060607

[22] Dhawan S, Singla AK, Sinha VR (2004) Evaluation of mucoadhesive properties of Chitosan Microspheres prepared by different methods. AAPS Pharma Sci Tech 5: 1–7. 10.1208/pt050467 PMC2750492 15760064

[23] Shelma R, Sharma CP (2011) Submicroparticles composed of amphiphilic chitosan derivative for oral insulin and curcumin release applications. Colloids and Surf B: Biointerfaces 88: 722–728. 21893399 10.1016/j.colsurfb.2011.08.007

[24] Dole VP (1956) A relation between non esterified-fatty acids in plasma and the metabolisms of glucose. J Clin Invest 35: 150–154. 13286333 10.1172/JCI103259 PMC438791

[25] Condrea E, Mammon Z, Aloof S, Devris A (1964) Susceptibility of erythrocytes of various animal species to the hemolytic and phospholipid splitting action of snake venom. Biochim. Biophys Acta 84: 365–75. 10.1016/0926-6542(64)90001-0 14230811

[26] Kondo H, Kondo S, Ikegawa H, Murata R, Oshaka A (1960) Studies on the quantitative method for determination of hemorrhagic activity of Habu snake venom. Jpn J Med Sci Biol 13: 43–51. 13853435 10.7883/yoken1952.13.43

[27] SaÂnchez EF, MagalhaÄ es A, Mandelbaum FR, Diniz CR (1991) Purification and characterization of the hemorrhagic II factor from the venom of the bushmaster snake (Lachesis muta muta). Biochim Biophys Acta 1074: 347–354. 1909578 10.1016/0304-4165(91)90084-t

[28] Theakston RDG, Reid HA (1979) Enzyme-linked immunosorbent assay (ELISA) in assessing antivenom potency. Toxicon 17: 511–515. 516083 10.1016/0041-0101(79)90284-8

[29] Clemens R, Pukrittayakamee S, Vanijanonta S, Nontprasert A, Bock HL, et al (1995) Therapeutic effects of antivenom supplemented by antithrombin III in rats experimentally envenomated with Russell's viper (*Daboia russelli siamensis*) venom. Toxicon 33 1: 77–82. 7778131 10.1016/0041-0101(94)00131-q

[30] Mukhopadhyay P, Mishra R, Rana D, Kundua PP (2012) Strategies for effective oral insulin delivery with modified chitosan nanoparticles: A review. Prog Polym Sci 37: 1457–1475.

[31] Kinesh VP, Neelam DP (2010) Novel approaches for oral delivery of Insulin and current status of oral insulin products. IJPSN 3: 1057–1064.

[32] Simpson ID, Jacobsen IM (2009) Antisnake Venom Production Crisis—Who Told Us It Was Uneconomic and Unsustainable?. Wilderness & Environmental Medicine 20: 144–155. 19594208 10.1580/08-WEME-CON-273R1.1

[33] Hamman JH (2010) Chitosan Based Polyelectrolyte Complexes as Potential Carrier Materials in Drug Delivery Systems. Mar Drugs 8: 1305–1322. 20479980 10.3390/md8041305 PMC2866488

[34] Lin YH, Linang HF, Chung CK, Chen MC, Sung HW (2005) Physically crosslinked alginate/N, O-carboxymethyl chitosan hydrogels with calcium for oral delivery of protein drugs. Biomaterials 26: 2105–2113. 15576185 10.1016/j.biomaterials.2004.06.011

[35] Tavakol M, Vasheghani-Farahani E, Hashemi-Najafabadi S (2013) The effect of polymer and CaCl2 concentrations on the sulfasalazine release from alginate-N,Ocarboxymethyl chitosan beads. Progress in biomaterial 2: 10. 10.1186/2194-0517-2-10 PMC5151116 29470666

[36] Shi J, Alves NM, Mano JF (2006) Drug Release of pH/Temperature-Responsive Calcium Alginate/Poly(N-isopropylacrylamide) Semi-IPN Beads. Macromol Biosci 6: 358–363. 16671051 10.1002/mabi.200600013

[37] Setterstrom JA, Tice TR, Meyers WE, Vincent JW (1984) Development of encapsulated antibiotics for topical administration to wounds, in: Second World Congress on Biomaterials 10th Annual Meeting of the Society for Biomaterials, Washington, DC, April 27–May 1, p. 4.

[38] Allamneni Y, Reddy BVVK, Chary PD, Rao NVB, Kumar SC, et al (2012) Performance Evaluation of Mucoadhesive Potential of Sodium Alginate on Microspheres Containing an Anti-Diabetic Drug: Glipizide. IJPSDR 4: 115–122.

[39] World Health Organization (1981) Progress in the Characterization of Venoms and Standardization of Antivenoms. WHO Offset Publication No. 58. World Health Organization, Geneva. 7245916

[40] Ownby CL, Odell GV (1983) Pathogenesis of skeletal muscle necrosis induced by tarantula venom. Exp Mol Pathol 38: 283–296. 6852204 10.1016/0014-4800(83)90069-2

[41] da Silva MH, Bie OG (1982) Titration of antiserum to South American rattlesnake (Crotalus durissus terrificus) venom by measuring inhibition of phospholipase A2 activity. Toxicon 20: 563–569. 7101307 10.1016/0041-0101(82)90050-2

[42] Gutierrez JM, Avila C, Rojas E, Cerdas L (1988) An alternative *in vitro* method for testing the potency of the polyvalent antivenom produced in Costa Rica. Toxicon 26: 411–413. 3406951 10.1016/0041-0101(88)90010-4

[43] Boche RD, Russell FE (1968) Passive hemaggglutination studies with snake venom and anti venom. Toxicon 6: 125–130. 5721657 10.1016/0041-0101(68)90031-7

[44] Yau TW, Kuchel RP, Koh JM, Szekely D, Mirtschin PJ, et al (2012) Cytoskeletal rearrangements in human red blood cells induced by snake venoms: light microscopy of shapes and NMR studies of membrane function. Cell Biol Int 36: 87–97. 21933154 10.1042/CBI20110012

[45] Rungsiwongse J, Ratanabanangkoon K (1991) Development of an ELISA to assess the potency of horse therapeutic antivenom against Thai cobra venom. J Immunol Methods 136: 37–43. 1995711 10.1016/0022-1759(91)90247-d

[46] Isbister GK (2010) Antivenom efficacy or effectiveness: The Australian experience. Toxicology 268 3: 148–54. 19782716 10.1016/j.tox.2009.09.013

[47] Matsuno T, Hashimoto Y, Adachi S, Omata K, Yoshitaka Y, et al (2008) Preparation of injectable 3D-formed *β*-tricalcium phosphate bead/alginate composite for bone tissue engineering. Dental Materials Journal 27 6: 827–834. 19241692 10.4012/dmj.27.827

[48] Segura A, Herrera M, González E, Vargas M, Solano G, et al (2009) Stability of equine IgG antivenoms obtained by caprylic acid precipitation: towards a liquid formulation stable at tropical room temperature. Toxicon 53 6: 609–15. 19673074 10.1016/j.toxicon.2009.01.012

